# A pathway linking reward circuitry, impulsive sensation-seeking and risky decision-making in young adults: identifying neural markers for new interventions

**DOI:** 10.1038/tp.2017.60

**Published:** 2017-04-18

**Authors:** H W Chase, J C Fournier, M A Bertocci, T Greenberg, H Aslam, R Stiffler, J Lockovich, S Graur, G Bebko, E E Forbes, M L Phillips

**Affiliations:** 1 Department of Psychiatry, University of Pittsburgh School of Medicine, Pittsburgh, PA, USA

## Abstract

High trait impulsive sensation seeking (ISS) is common in 18–25-year olds, and is associated with risky decision-making and deleterious outcomes. We examined relationships among: activity in reward regions previously associated with ISS during an ISS-relevant context, uncertain reward expectancy (RE), using fMRI; ISS impulsivity and sensation-seeking subcomponents; and risky decision-making in 100, transdiagnostically recruited 18–25-year olds. ISS, anhedonia, anxiety, depression and mania were measured using self-report scales; clinician-administered scales also assessed the latter four. A post-scan risky decision-making task measured ‘risky' (possible win/loss/mixed/neutral) fMRI-task versus ‘sure thing' stimuli. ‘Bias' reflected risky over safe choices. Uncertain RE-related activity in left ventrolateral prefrontal cortex and bilateral ventral striatum was positively associated with an ISS composite score, comprising impulsivity and sensation-seeking–fun-seeking subcomponents (ISSc; *P*⩽0.001). Bias positively associated with sensation seeking–experience seeking (ES; *P*=0.003). This relationship was moderated by ISSc (*P*=0.009): it was evident only in high ISSc individuals. Whole-brain analyses showed a positive relationship between: uncertain RE-related left ventrolateral prefrontal cortical activity and ISSc; uncertain RE-related visual attention and motor preparation neural network activity and ES; and uncertain RE-related dorsal anterior cingulate cortical activity and bias, specifically in high ISSc participants (all ps<0.05, peak-level, family-wise error corrected). We identify an indirect pathway linking greater levels of uncertain RE-related activity in reward, visual attention and motor networks with greater risky decision-making, via positive relationships with impulsivity, fun seeking and ES. These objective neural markers of high ISS can guide new treatment developments for young adults with high levels of this debilitating personality trait.

## Introduction

Impulsive sensation seeking (ISS) is a personality trait comprising the component traits of impulsivity, behavior characterized by little or no forethought, reflection or consideration of the consequences, and often prematurely elicited;^1^ and sensation seeking, the tendency and willingness to seek, and take risks for, novel and intense sensations and experiences.^2^ Several well-validated self-report scales are used to measure ISS. These include the UPPS-P Impulsive Behavior Scale,^3^ reflecting impulsivity subcomponents such as non-planning, lack of perseverance, sensation seeking, positive and negative urgency. The latter two components reflect impulsive behavior that is determined by the current (positive or negative) emotional state. The Barratt Impulsiveness Scale (BIS-11)^4^ incorporates standardized measures of impulsivity subcomponents: non-attention; non-planning; and motor impulsivity. The Behavioral Activation System scale (BAS^5^) reflects reward sensitivity and sensation-seeking subcomponents: drive; fun seeking; and reward responsivity. The Zuckerman Sensation Seeking Scale (SSS^6^) includes standardized measures of sensation-seeking subcomponents: experience seeking; thrill seeking; disinhibition; and boredom susceptibility. ISS peaks in adolescence and early adulthood,^7, 8^ and can have deleterious consequences, for example, risky decision-making and behaviors, poor social and occupational function, accidental injury and death.^9^ Notably, the persistence of high levels of disadvantageous impulsivity and sensation-seeking traits through to, and during, adulthood distinguishes high trait ISS from the normal adaptive changes in affective processing, inhibitory control and cognitive flexibility observed in adolescence.^9^

Different ISS subcomponents are associated with different measures of poor psychosocial outcome. For example, high levels of positive and negative urgency are associated with development of a range of different psychopathology,^10^ while positive urgency is associated with response inhibition deficits.^11^ Both sensation seeking and positive urgency are also associated with development of bipolar disorder (BD^12, 13, 14, 15, 16^). For example, BAS scores are significantly higher in BD than healthy individuals,^13, 17, 18, 19^ as are scores on other measures of sensation seeking^20^ and positive urgency.^21^ There is also increasing evidence that in young adulthood, the peak age-range for emergence of many major psychiatric disorders,^22^ high ISS may be a risk factor for BD: BAS scores are positively associated with, and account for 27% of, current mania severity in young adults with subthreshold symptoms who are at risk for future BD.^15, 23^ In addition, higher BAS scores are associated with a threefold greater probability of future BD in adolescents and young adults.^24^ High levels of sensation seeking are also associated with development of substance use and substance use disorders, and other risky behaviors.^25^ Interestingly, there is further evidence suggesting a moderating effect of impulsivity on positive relationships between measures of sensation seeking and substance use disorders in young adults,^26^ where greater sensation seeking is positively associated with higher levels of substance use disorders in high, but not low, impulsive individuals.

Identifying neural markers of high ISS in young adulthood, and pathways linking these markers with deleterious risky decision-making, can thus provide objective biomarkers to guide interventions for young adults with high ISS who have not yet developed BD, but who are at risk for the disorder and the other deleterious psychosocial consequences described above, as well as facilitating new treatment developments for BD. Furthermore, identifying neural measures of high ISS in young adulthood, a critical developmental period when brain development is still occurring,^27, 28, 29^ allows for subsequent neurobiological interventions to take advantage of the plasticity of the brain during this developmental period to minimize, or even prevent, long-term abnormalities in neural circuitry and chronic mental health problems associated with high ISS.

Previous studies demonstrated relationships between different ISS subcomponents and neural activity during a variety of tasks in healthy adults as yet unaffected by psychiatric illness.^30, 31, 32^ However, few studies have examined tripartite relationships among ISS subcomponents, underlying neural activity and predisposition to risky decision-making and behaviors in young adults. One such study showed a mediating effect of negative urgency on the relationship between amygdala and ventrolateral prefrontal cortical activity to emotional cues and risk taking.^33^ Nevertheless, pathways linking neural activity, high ISS and risky decision-making in young adults remain poorly understood.

One of the constructs of the positive valence system domain of the National Institute of Mental Health Research Domain Criteria (RDoC) that may be especially relevant to several ISS subcomponents is approach motivation-reward expectancy (RE), which describes the impact of the expectation of rewarding outcomes. This construct may, for example, be relevant to understanding positive urgency, given that during uncertain RE, impulsive thoughts and decisions may be triggered in response to positive emotional states. One promising way forward in the search for neural markers of ISS subcomponents is thus to employ reward paradigms that include an uncertain RE condition to determine the extent to which different ISS subcomponents are associated with distinct patterns of reward circuitry activity in uncertain future reward contexts. We have used such a reward paradigm in previous neuroimaging studies in youth and adults, where on each trial, the expectancy of possible reward phase is distinct from the outcome phase.^34^

We previously reported associations between greater levels of a sensation-seeking subcomponent, the fun-seeking subscale of the BAS and greater activity in ventral striatum (VS) during uncertain RE across healthy adults and adults with different types of BD.^35^ Other studies reported positive associations between VS activity during RE and higher levels of positive arousal (including fun seeking);^36^ greater lateral prefrontal cortical (extending into ventrolateral prefrontal cortex, vlPFC) activity in high ISS vs low ISS adolescents to wins versus non-wins;^37^ elevated VS activity during RE in high versus low impulsive adults^38^), and in adults with high versus low reward sensitivity, as measured by the BAS;^39^ as well as a positive association between amygdala and ventral anterior cingulate cortical activity during expectancy of reward and impulsivity.^40^ We also reported greater activity in left vlPFC and VS during uncertain RE in individuals with BD type I (BDI) in remission.^41^ Similarly, other studies reported abnormally elevated left vlPFC activity to reward in healthy youth at risk of BD,^42^ and in mania;^43^ and elevated VS activity during RE in adults with bipolar disorder.^44^ Together, these studies suggest that neural regions most commonly associated with different ISS subcomponents during reward expectancy may include left vlPFC and VS. The VS encodes reward anticipation and outcome events, often in a manner predicted by the temporal difference model.^45^ The vlPFC links cues to specific reward outcomes.^46, 47^ Greater activity in left vlPFC and VS during uncertain RE may thus reflect greater encoding of cue-outcome associations in potentially rewarding contexts, and may be a transdiagnostic neural marker of high levels of ISS and disorders such as BD that are characterized by elevated ISS. The left laterality of vlPFC response may reflect the left frontal cortex's role in approach behaviors.^48^ These findings also suggest that abnormally elevated uncertain RE-related activity frontostriatal reward circuitry, specifically in left vlPFC and VS, in young adults with high ISS may predispose to risky decision-making and associated behaviors, but this remains unexamined.

We aimed, in a large participant sample: (1) to determine relationships between different ISS subcomponents, and activity in left vlPFC and bilateral VS during uncertain RE in 18–25-year olds recruited transdiagnostically. (2) To determine relationships among ISS subcomponents and risky decision-making. (3) To determine relationships among specific ISS subcomponents associated with uncertain RE-related activity in left vlPFC and bilateral VS, and ISS subcomponents associated with risky decision-making, to identify a potential direct or indirect pathway linking uncertain RE-related reward circuitry activity, ISS subcomponents and risky decision-making. We recruited distressed, treatment-seeking young adults and healthy young adults in the community, to sample participants across the ISS range. We had the following specific hypotheses:

Hypothesis (H1): During uncertain RE, greater activity in left vlPFC and bilateral VS to uncertain RE would be positively associated with higher levels of ISS across all participants. Findings from extant studies did not allow us to make hypotheses regarding the specific ISS subcomponents that would be related to uncertain RE-related activity.

H2: There would be positive correlations between higher levels of ISS, especially sensation-seeking subcomponents, for example, experience and fun seeking and greater post-scan risky decision-making, given previous findings linking high levels of sensation seeking with risky decision and behaviors.^49^

H3. Given previous findings showing a moderating effect of impulsivity on the association between sensation seeking and risky decision-related substance use disorders in young adults,^26^ we hypothesized that any ISS impulsivity subcomponents identified when testing H1 would moderate any positive relationships between sensation-seeking subcomponents and risky decision-making identified when testing H2.

## Materials and methods

### Experimental design: participants

Fifty-three 18–25-year olds who were actively seeking help for psychological distress, irrespective of diagnosis, in the Pittsburgh area were recruited via community advertisement and student counseling services. Fifty-six healthy 18–25-year olds with no previous personal or family history of psychiatric illness in first-degree relatives were recruited via community advertisement and a participant registry in Pittsburgh. All participants were right-handed and English speaking. Demographic information (age, gender and years of education) were documented for all participants (Table 1). Exclusion criteria are in the Supplementary Materials. The participant population reflected the demographics of Pittsburgh and the surrounding area. The study protocol was approved by the University of Pittsburgh Institutional Review Board. After complete description of the study to the individuals, written informed consent was obtained. Two participants were excluded due to excessive motion (>5 mm), one participant was excluded due to excessive task performance errors (20, all other participants <12) and six participants were excluded for excessive signal loss in right VS, left VS or bilateral amygdala (>30%). The final sample comprised 48 distressed individuals and 52 healthy controls.

### Behavioral trait and clinical measures

To assess ISS component traits of impulsivity and sensation seeking, and their respective subcomponents, participants completed the following self-report scales (Table 1): the Zuckerman SSS;^6^ the Behavioral Inhibition and Activation System Scales^5^ (BIS/BAS); the Barratt Impulsiveness Scale^4^ (BIS-11); and the UPPS-P Impulsive Behavior Scale.^3^ Other traits that could impact reward circuitry activity, namely, anhedonia and anxious arousal, were measured using the Snaith-Hamilton Pleasure Scale^50^ (SHAPS), the Mood and Anxiety Symptom Questionnaire—Anhedonia Scale^51^ (MASQ-AD) and Anxious Arousal Scale^51^ (MASQ-AA), and the State-Trait Anxiety Inventory^52^ (STAI). To measure depression and mania symptom severity, the study clinician administered the Hamilton Rating Scale for Depression^53^ (HAMD) and the Young Mania Rating Scale^54^ (YMRS) to all participants. Current anxiety was measured using the clinician-administered Hamilton Anxiety Rating Scale^55^ (HAMA).

### fMRI data acquisition parameters

Functional neuroimaging data were collected using a 3.0 Tesla Siemens Trio 2 MRI scanner at the University of Pittsburgh. Blood-oxygenation-level-dependent (BOLD) images were acquired with a multi-band gradient echo EPI sequence (18 slices, three-factor multiband; 2.3 mm isotropic voxels; TR·TE=1500/30 ms; field of view=220 × 220 mm; matrix 96 × 96; flip angle 55°, bandwidth 1860 Hz Px^–1^). Structural 3D axial MPRAGE images were acquired in the same session (TR·TE=1500/3.19 ms; flip angle 8° FOV=256 × 256 mm; 1 mm isotropic voxels; 176 continuous slices), as were fieldmaps (2.3 mm isotropic voxels; TR=500 ms, TE1=4.92 ms, TE2=7.38 ms; FOV=220 × 220 mm; flip angle 45°, bandwidth 1302 Hz Px^–1^). Fieldmaps were not available for 11 participants (6 control, 5 distressed).

### fMRI paradigm

**Figure 1:**
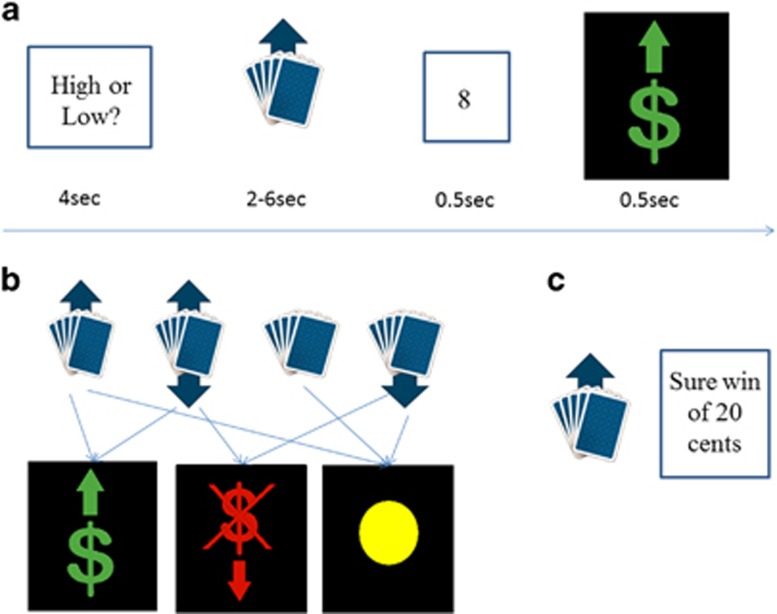
Reward paradigm design. (**a**) Trial structure demonstrating choice phase, anticipation phase, numerical feedback and outcome (win, loss and neutral). (**b**) Description of the outcomes associated with each of the four stimuli (win, mixed, neutral and loss respectively). Transition probabilities are 0.5 except for the neutral stimulus. (**c**) Example of a post-task trial, in which participant has to choose between card stimulus and ‘sure thing' option.

We employed a 16-min event-related card-guessing game adapted from previous studies^56, 57^ (Figure 1) to examine neural activity during anticipation and receipt of monetary reward. During each trial, individuals guessed via button press whether the value of a visually-presented card was high or low (4 seconds: presentation of a question mark). An expectancy cue was then presented for 2–6 seconds (jittered), with four types of cues/trial types described below. The outcome then appeared for 1 second (the number for 500 ms and then the feedback arrow for 500 ms), followed by a 0.5–1.5 second inter-trial interval. Individuals practiced the task before the scan. The four trial types were as follows: expectation of possible win, followed by win outcome (win trials) or no change (disappointment trials); expectation of possible loss, followed by loss (loss trials) or no change (relief trials); mixed win/loss trials, followed by win or loss; neutral trials, followed by no change. The paradigm was administered in 2, 8 minute blocks, with 48 trials per block: 12 trials each for each trial type; and 50% chance of each outcome. Trials were presented in a random order with predetermined outcomes. Individuals were told that their performance would determine a monetary reward after the scan: $1 for each win and 75 cents deducted for each loss. Total possible earnings were $6.

### Data analyses: first level (participant level) neuroimaging data analysis

Data were preprocessed using a combination of software packages (SPM, FSL, AFNI) implemented in Nipype.^58^ Data for each participant were realigned to the first volume in the time series to correct for head motion. Realigned BOLD images were then co-registered with the subject's anatomical image. Distortion was of this image was corrected with a fieldmap, employing the FSL FUGUE package. The anatomical image was normalized to the MNI/ICBM 152 template using a non-linear transformation and segmented into separate tissue types. BOLD images were then transformed to the same space via the segmented structural image (the DARTEL method), at a resolution of 2 mm^3^ isotropic voxel size. BOLD images were corrected for activity spikes using the AFNI 3dDespike tool, normalized for intensity and then spatially smoothed with a FWHM of 6 mm, using FSL's SUSAN adaptive smoothing method.

### Statistical analysis

#### *First level (participant level) neuroimaging data analysis*

A first-level fixed-effect general linear model (GLM) was constructed for each participant using Statistical Parametric Mapping software, Version-8 (SPM8). The regressor of primary interest was RE, a parametric modulator coupled to the 2–6 s duration anticipation period, which reflected the expected value of the arrow. It was set to +0.5 for the possible win condition (50% chance of winning $1), –0.375 for the possible loss condition (50% chance of losing $0.75), +0.125 for the mixed condition (50% chance of winning $1; 50% chance of losing $0.75), and zero for the neutral condition. The three main regions of interest (ROIs) were left and right ventral striatum ROIs, and the left vlPFC (Supplemental Materials).

Two additional regressors of secondary interest were included in the first-level model: uncertain outcome expectancy (OE) and prediction error (PE). The OE regressor was coupled to the anticipation period and reflected the range of the (unsigned) value of possible outcomes. This measure is greatest for the mixed trials ($1−$0.75 = 1.75), lowest for neutral trials (zero), and intermediate for possible win ($1−$0 = 1) and possible loss (0−$0.75 = 0.75) trials. Gram-Schmidt orthogonalization was applied as is standard in SPM, with the RE regressor preceding the OE regressor. The PE regressor, coupled to the outcome, was determined by the difference between the outcome and the EV, that is, +0.5 for a win and –0.5 for no win in the possible win condition, +0.375 for a no loss and –0.375 for a loss in the possible loss condition, +0.875 for a win and –0.875 for a loss in the mixed condition and zero in the neutral condition. Another regressor was included to model omission errors, if these were made. Given that there were two blocks of the task, this GLM was fit to each block separately, and the parameter estimates for a given effect type were combined across each.

Follow-up analyses were performed to confirm the trial types that contributed to the findings. We used a separate first-level model in which possible win anticipation and possible loss anticipation trials were contrasted, respectively, with neutral anticipation trials. In this design, each of the four anticipation conditions were modelled separately, but the outcome (PE) condition was identically modelled (Supplemental Information for findings regarding these contrasts).

To create the GLM from the design, the canonical hemodynamic response function was convolved with each regressor. Movement parameters from the realignment stage were entered as covariates of no interest to control for participant movement. A regressor to correct for physiological fluctuations was included, derived from the mean signal within white matter, cerebrospinal fluid and high temporal standard deviation voxels.^59, 60^ A high-pass filter (60 s), and autoregressive (AR(1)) modelling were also implemented during first-level model fitting.

#### Post scan risky decision making task

Following the card guessing task, participants performed a decision-making task outside of the scanner. Participants made a choice between one of the four card stimuli to which they were exposed during the task (win/loss/mixed/neutral), with the above chances of winning or losing, for example, 50% chance of winning $1 and 50% chance of no change for the card associated with the possible win expectancy condition, and one of several ‘sure thing' option cards. The latter ranged from 100% winning $0.80 to 100% losing $0.80 in $0.10 intervals (4 stimuli by 17 levels=68 trials total), and the amount and probability (that is, 100% each time) were presented explicitly. The pattern of choices for the cards or the sure thing options were modelled, and a ‘bias' parameter was derived, which reflected the preference for risky wins (see Supplemental Materials for further detail).

#### Second level neuroimaging data analyses testing hypotheses

**H1: uncertain RE-related neural activity and ISS**

Due to the number of correlated predictors, we employed elastic net regression (‘lasso' function implemented in MATLAB; see Supplementary Information) to identify the relationship between ISS component trait measures and RE-related bilateral VS and left vlPFC activity in the ROIs described above. Elastic net is a modified form of least squares regression that penalizes complex models with regularization parameters (λ1, λ2)^61, 62^ and is sensitive to correlated variables.^62^ The regularization (lasso/ridge regression) parameters shrink coefficients toward zero, and eliminate unimportant terms entirely.^61, 63, 64^ Cross validation identifies the optimal penalty terms that minimizes mean cross validated error, reduce the chances of overfitting and enforces recommended sparsity in the solution.^61^ Elastic net regression models thereby allow inclusion of a relatively large number of correlated independent variables in regression models. The main independent variables were all ISS component traits, including all impulsivity and sensation-seeking subcomponent measures (Table 1). This regression model also included the following covariates: state anxiety; depression severity (HAMD); anhedonia; group (distressed, healthy); demographic variables (age, gender and years of education); and motion (framewise displacement^65^). Mania was not included as a covariate as only one participant scored >10 on the YMRS. Dependent variables were extracted BOLD signal from left vlPFC and bilateral VS during uncertain RE. Three separate elastic net models were run, one for each of these three ROI activity-dependent variables.

A test statistic for elastic net models is still under development.^66^ Thus, to provide significance levels of main identified independent variable-dependent variable relationships, we ran multiple regression models in which independent variables were those (non-zero) ISS variables associated with mean RE-related activity in the three ROIs, left vlPFC and bilateral VS and at least one of those regions independently (the latter to confirm its relevance). To avoid inclusion of highly correlated independent variables and covariates, we combined, in these multiple regression models, impulsivity and sensation-seeking subcomponents associated with ROI RE-related activity in the above elastic net analysis (see Supplementary Information). Covariates were group, motion and demographic variables; other clinical variables emerging in the elastic net analysis were assessed independently.

**H2: ISS and risky decision-making**

To test relationships between ISS and risky decision-making, we used a similar elastic net model as for H1, using as independent variables all ISS component trait and subcomponent measures, and covariates as above (apart from scanner motion). The dependent variable was bias, our main measure of risky decision-making, as described above. To provide significance levels of main identified independent variable-dependent variable relationships, we ran a multiple regression model in which independent variables were all ISS subcomponents associated with bias as identified by the elastic net analysis, and the dependent variable was bias. Covariates used in this multiple regression model were group and demographic variables.

**H3: relationship among H1 ISS component traits, H2 ISS component traits and risky decision-making**

We followed up the hypothesis tests of H1 and H2, described above, by running a multiple regression analysis, including as the main independent variables the interaction term between the composite of the ISS subcomponents associated with RE-related neural activity in H1 and the composite of the ISS subcomponents associated with bias in H2, as well as their respective main effects. The dependent variable was bias, while additional covariates were group and demographic variables.

### Exploratory analyses

We used a whole-brain multiple regression model in SPM, to identify any additional neural regions, unexamined by the above ROI approach, in which uncertain RE-related activity showed relationships with ISS subcomponents. Independent variables were ISS subcomponent independent variables that were identified as part of H1 and H2 testing using the elastic net analysis. We used a similar SPM whole-brain multiple regression model to identify neural regions in which uncertain RE-related activity showed direct relationships with post-scan risky decision-making (bias). In all of these whole-brain analysis, in addition to the primary variables describe above, covariates were group, motion and demographic variables.

As described above, whole-brain analyses were preformed to identify patterns of neural activity associated with the uncertain OE and PE regressors. In addition, follow-up whole-brain analyses were performed to check the direction of the findings regarding patterns of neural activity associated with the uncertain RE regressor, using a separate first-level model in which possible win anticipation and possible loss anticipation trials were contrasted, respectively, with neutral anticipation trials. For all exploratory analyses, we used a peak-level, family-wise error corrected *P*<0.05. Additional findings at uncorrected thresholds (*P*<0.001, 20 voxel cluster) are reported for completeness (see Supplementary Tables S2–S8).

## Results

### Neuroimaging data analyses

#### H1: uncertain RE-related neural activity and ISS

Four elastic net regression models were run, modeling RE-related activity extracted from left vlPFC, and bilateral VS and the mean of all three regions (Table 2). These analyses yielded five ISS subcomponents that predicted both mean RE-related activity across all regions and in at least one of the regions independently: BIS-11 motor and attentional subscales, UPPS-P Positive and Negative Urgency and BAS fun seeking. In addition, group, HAMD depression severity, STAI trait anxiety and motion were also identified.

**Figure 2:**
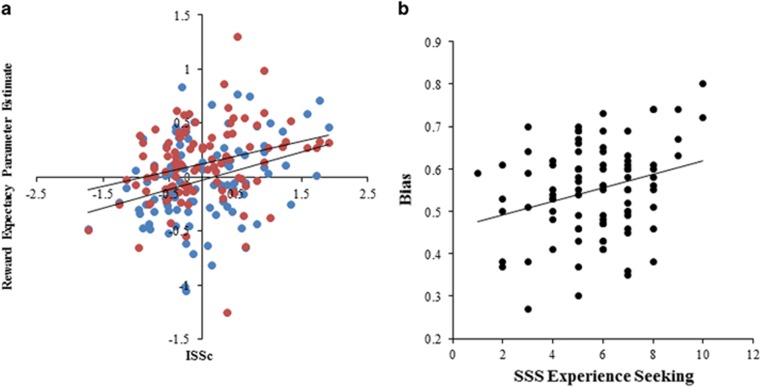
(**a**) Associations between ISSc with left vlPFC (blue; *r*=0.32, *P*=0.001) and left VS (red; *r*=0.28, *P*=0.005). (**b**) Association between SSS ES and bias score (*r*=0.29, *P*=0.006). These figures show relationships without the addition of covariates. The relationships remained significant with the addition of covariates (see Results). ES, experience seeking; ISSc, impulsive sensation seeking subcomponents; SSS, Sensation Seeking Scale; vIPFC, ventrolateral prefrontal cortex; VS, ventral striatum.

Given that the ISS five measures were positively correlated with each other (Supplementary Table S9), we combined these impulsivity subcomponents into a single variable (ISS composite: ‘ISSc') by *z*-transforming each and averaging them. Multiple regression analysis including covariates: group; demographic variables; and motion, supported the hypothesized relationship between ISSc and RE-related activity in left vlPFC, left VS and right VS (Table 2 and Figure 2a). These models were also accompanied by significant effects of group in the left and right VS, with distressed individuals showing lower RE-related activity than controls. Although identified by the elastic net, motion was not significantly associated with RE-related activity in any ROI within the multiple regression models. Trait anxiety and depression severity were also identified within the elastic net, and were inversely associated with RE-related activity in right and left VS (t's=-2.61 to -3.35, p's=0.011 to 0.001), but only if group was not also included in the model. In other words, these variables did not explain substantial further variance beyond that explained by group, and were not considered further. Finally, combining RE-related activity in all three neural regions into a single neural activity variable led to significant effects of ISSc and group when the above covariates were included. Overall, none of the demographic covariates played an important role, such that the significance of the group (in VS alone) and ISSc effects were comparable if the other covariates were not included (Table 2).

#### H2: ISS and risky decision-making

Nine participants had high beta scores and poor model fits, forming a separate distribution and were excluded from data analysis (Supplementary Materials). This left 91 participants for analysis of decision-making bias. Elastic net analysis was performed with all ISS component trait and subcomponent measures and covariates as in HI elastic net models (aside from motion) as predictors of bias. Only one variable was identified: the experience seeking scale of the SSS (ES; exponent=0.015). With demographic variables as covariates, the ES-bias relationship was significant (*t*=3.018, *β*=0.31, *P*=0.003) and was similarly significant without covariates (*t*=2.83, *β*=0.29, *P*=0.006; Figure 2b).

#### H3. Relationship among H1 ISS impulsivity component traits, H2 ISS component traits and risky decision-making

ISSc was not related to bias (*P*>0.9) but was weakly related to SSS ES (*r*=0.24, *P*=0.021) in the sample with reliable decision-making data (*n*=91). There was, however, a significant ES by ISSc interaction on bias: when including covariates (*t*=2.68, *β*=0.28, *P*=0.009); without covariates (*t*=3.32, *β*=0.32, *P*=0.001). This interaction reflected a strong positive relationship between ES and bias in high (ISSc *z-*score greater than zero: *r*=0.57, *n*=39, *P*<0.001), but not low (ISSc z-score zero or less: *P*>0.47, *n*=52) ISSc participants.

### Exploratory analyses

**Figure 3:**
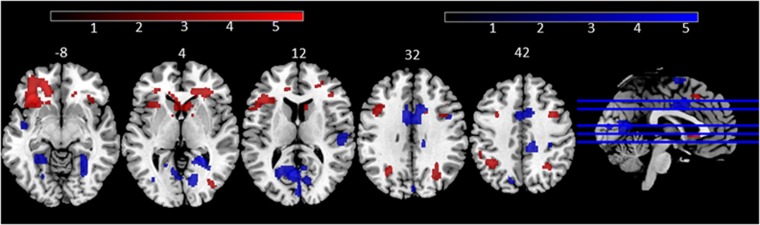
Distinct patterns of association between reward expectancy (RE) and ISSc (red), and RE and SSS ES (blue). Both maps thresholded at *P*<0.001 uncorrected, *k*=20 for display purposes. ES, experience seeking; ISSc, impulsive sensation seeking subcomponents; SSS, Sensation Seeking Scale. Scales reflect T statistics; numbers above axial slices reflect z coordinate of slice.

ISSc was positively associated with significant RE-related activity predominantly in left vlPFC, specifically in left frontal operculum, at corrected significance levels (Figure 3a and Supplementary Table S2). Distressed individuals showed significantly reduced activity compared to healthy individuals, mainly in the left frontal operculum (Supplementary Table S5). By contrast, SSS ES was associated with significant RE-related activity in occipital and premotor cortices (Figure 3b and Supplementary Table S3). There was no significant interaction of ISSc and SSS ES, and no significant main effect of bias, on whole-brain RE-related activity. Given the moderation by ISSc of the positive SSS ES-bias relationship, however, we wished to determine if there were differential patterns of RE-related whole-brain activity to bias in high versus low ISSc participants (subgroups differentiated by a split of ISSc scores, as above). There was a significant effect of bias in high ISSc participants on RE-related activity in dorsal anterior cingulate cortex (Supplementary Table S4). There was no significant RE-related whole-brain activity in low ISSc participants at corrected thresholds.

To determine whether patterns of neural activity were specific to uncertain RE rather than common to uncertain OE, we performed analyses examining neural activity to uncertain OE. No relationships between ISSc or SSS ES and uncertain OE-related whole-brain activity were seen at corrected thresholds, and only minor differences at uncorrected thresholds.

We also examined the possibility that the effect of ISSc on RE-related activity may have been related to enhanced deactivation to loss anticipation cues, rather than enhanced activity to win anticipation cues. This required that we considered the neutral anticipation cue as the baseline, and contrasted this with the possible loss anticipation and possible win anticipation cues, respectively. Although not perfectly controlled for uncertainty, the results from this analysis favored an interpretation in terms of heightened reward anticipation rather than reduced loss anticipation: increasing ISSc score was associated with increased win anticipation-related versus neutral condition-related activity in a similar set of regions as the RE regressor, with one peak approaching corrected significance (Supplementary Table S6). By contrast, the neutral versus loss anticipation contrast yielded no significant associations with ISSc, while the minor differences seen at uncorrected thresholds were not in directly relevant regions.

## Discussion

To our knowledge, we show for the first time a pathway linking uncertain RE-related activity, ISS and risky decision-making in young adults. First, uncertain RE-related activity in left vlPFC and bilateral VS was significantly associated with specific ISS components related to both impulsivity and sensation seeking, positive and negative urgency, BIS Motor and Attention, and BAS fun seeking. Second, a specific measure of sensation seeking, SSS ES, was positively associated with risky decision-making (bias). Third, the positive relationship between ES and bias was moderated by the above composite measure of ISS, such that, the positive relationship between ES and bias was evident only in individuals with high levels of this ISS composite.

Our findings accord with previous data associating greater striatal activity during RE with greater impulsivity,^67, 68^ which may be mediated by increased striatal dopamine release.^69, 70^ Our finding of a positive association between an ISS composite score (ISSc) and uncertain RE-related left vlPFC and VS activity also links previous findings of elevated activity in these regions during uncertain RE in individuals with BD.^35, 41^ Thus, our present findings highlight urgency and attentional/motor impulsivity as ISS subcomponents that may have contributed to previous findings of elevated uncertain RE-related VS and left vlPFC activity in individuals with BD. Our findings also parallel our previous observation relating BAS fun-seeking to VS activity during uncertain RE across healthy individuals and individuals with bipolar disorder.^35^ In addition, our findings not only support previous reports of significant contributions of sensation seeking to outcomes associated with risky decision-making,^71, 72, 73^ but also support previous findings showing a moderating effect of impulsivity on the relationship between sensation seeking and risky decision-making, such that that high levels of both impulsivity and sensation seeking are necessary for risky decision-making.^26^ Importantly, our findings highlight the importance of the left vlPFC in encoding stimulus-outcome associations,^46^ optimistic bias,^74^ free choice^75^ and approach-related emotions^48^ and show more precisely how elevated activity in bilateral VS and left vlPFC during uncertain RE predisposes to risky decision-making via associations with high levels of impulsivity, fun seeking and sensation seeking.

Exploratory whole-brain analyses revealed significant positive associations between ISSc and activity in left vlPFC, particularly in the left frontal operculum, which contributes to flexible cognitive control and preparatory attention.^76, 77, 78^ A different network of whole-brain neural regions was associated with ES, particularly visual cortical regions and premotor cortex, which subserve visual attentional processing^79, 80, 81^ and motor preparation.^82^ One interpretation of these findings is thus that the regions associated with ISSc may relate to stimulus-outcome evaluation, while those associated with ES may reflect visual attention and motor processes associated with examination of rewarding versus less rewarding cues, and automatic motor preparation. These different processes may interact to determine risky decision-making, with ES associated with risk preference, and ISSc determining how rigidly risk preference is reflected in behavior. While there was no significant RE-related whole-brain activity associated with bias across all participants, in participants with high ISSc scores, there was a significant association between bias and RE-related activity in a region in dorsal anterior cingulate cortex (ACC). A similar region was also activated during RE across all participants, irrespective of ISSc score (Supplementary Information). Thus, the ACC appears to reflect the choice policy used in the high ISSc participants (i.e. high reward preference in the high bias participants: see also Paulus and Frank^83^). The relationship was not evident in the low ISSc participants, consistent with the notion that these individuals might use a mixture of choice policies.

Our additional analyses confirmed that the positive association between left vlPFC and bilateral VS activity and ISSc score observed to uncertain RE was specific to uncertain RE and not demonstrated to uncertain OE. Furthermore, additional analyses revealed that greater ISSc score was associated with greater possible win anticipation-related versus neutral condition-related activity in similar regions to those activated in response to the uncertain RE regressor, while this relationship was not observed to the loss anticipation versus neutral contrast. These findings indicate that the positive relationship observed between ISSc score and uncertain RE-related reward activity was specific to the context of possible future reward rather than to contexts of uncertainty *per se* or possible future loss.

In addition to the positive effect of ISSc on RE-related activity in the VS and left vlPFC, there was an effect of similar magnitude of group, with distressed individuals showing reduced RE-related activity in the VS regions compared to controls. Individual differences in depression severity and trait anxiety had a similar impact. This is consistent with a variety of studies of depressed individuals^84, 85, 86^ and bipolar disorder,^87^ although not two of our prior studies which used a similar card-guessing paradigm in unipolar (and bipolar) depressed individuals.^88, 89^ Two simple explanations for this latter discrepancy are, first, that this present paradigm includes more trials, which may allow RE-related activity to grow in strength (see Chase *et al.*^34^), and thus be a more sensitive measure. Second, the combination of group and ISSc explained more variance in RE-related activity than the two measures separately, particularly in the VS. This implies that collecting information about both psychological distress and ISS may enhance the efficiency of statistical modeling of individual differences in reward function.

A limitation of the study was inclusion of different diagnostic categories. There were no significant relationships between diagnostic categories and main neuroimaging or risky decision-making variables, however (Supplementary Information). Importantly, the transdiagnostic recruitment strategy enabled us to obtain a sample well suited to the examination of dimensional phenomena, and one in which medication confounds were minimal. Correcting for the small between-group gender ratio difference had no effect on the pattern of results.

To our knowledge, our study is the first to show an indirect pathway linking greater levels of uncertain RE-related activity in reward, flexible cognitive control and decision-making, visual attention and motor networks with greater risky decision-making, via positive relationships with impulsivity, fun seeking and ES. These objective neural markers of high ISS can guide new treatment developments for young adults with high levels of this debilitating personality trait. The importance of these findings is twofold. First, this is the first time, to our knowledge, that such a stepwise relationship between neural circuitry activity in uncertain future reward contexts, ISS subcomponents and risky decision-making has been demonstrated in any age group. Second, by identifying objective, proximal neural markers related to more distal, risky decision-making, we provide neural targets for new interventions to modulate, and even ameliorate, abnormalities at all three levels of this pathway, that is, neural circuitry, modifiable personality traits and risky decision-making and related behaviors. Our findings are thus an important step forward to identifying neural targets for novel treatments, for example, new neurostimulation interventions, to help reduce risky decision-making in young adults across a range of different psychiatric disorders.

## Figures and Tables

**Table 1:** Table describing the demographic and task-related data for the distressed and healthy control groups

	*Control*	*Distressed*	*Statistical comparison*
Gender^a^	29 F, 23 M	36 F, 12 M	*χ*^2^=4.057, *P*=0.044
Age^a^	21.30 (1.76)	22.041 (2.22)	*T*(89.67)=−1.85 (*P*=0.068)
Education^a^	5.31 (1.058)	5.33 (1.098)	*T*<1
STAI trait^a^	30.56 (5.19)	56.27 (11.25)	*T*(64.96)=−14.48, *P*<0.001
STAI state	28.50 (6.17)	48.25 (11.66)	*T*(70.089)=10.46, *P*<0.001
HAMA	0.56 (1.092)	13.00 (6.66)	*T*(48.59)=−3.39, *P*<0.001
HAMD^a^	0.75 (1.38)	15.81 (6.59)	*T*(50.83)=−15.52, *P*<0.001
YMRS	0.21 (0.46)	3.60 (3.13)	*T*(45.54)=7.10, *P*<0.001
NART IQ	108.87 (6.71)	107.081 (8.030)	*T*(98)=1.21, *P*=0.23
Framewise displacement^a^	0.19 (0.066)	0.20 (0.10)	*T*<1
Main Task RT	780.74 (214.70)	808.037 (267.49)	*T*<1
Post Task RT^b^	2272.10 (609.12)	2398.63 (1018.73)	*T*<1
Bias^b^	0.54 (0.11)	0.56 (0.11)	*T*<1
Beta^b^	0.30 (0.091)	0.34 (0.12)	*T*(89)=1.72, *P*=0.088
BIC^b^	−24.068 (6.42)	−26.18 (7.61)	*T*(89)=1.43, *P*=0.16
BIS-11 Motor^a^	21.81 (3.087)	21.17 (4.27)	*T*<1
BIS-11 Attention^a^	14.40 (3.11)	17.75 (3.74)	*T*(98)=−4.88, *P*<0.001
BIS-11 Non Planning^a^	21.94 (4.22)	22.65 (5.00)	*T*<1
BAS Drive^a^	11.40 (1.88)	11.25 (2.65)	*T*<1
BAS Fun Seeking^a^	12.54 (1.75)	12.02 (2.56)	*T*(82.40)=1.17, *P*=0.25
BAS Reward responsiveness^a^	17.38 (1.85)	17.06 (2.046)	*T*<1
MASQ-AD^a^	50.63 (9.00)	75.48 (16.032)	*T*(72.67)=−9.45, *P*<0.001
MASQ-AA	18.25 (1.61)	29.98 (11.93)	*T*(48.58)=−6.75, *P*<0.001
SHAPS^a^	19.02 (5.11)	27.65 (6.88)	*T*(81.95)=−6.66, *P*<0.001
SSS Boredom Susceptibility^a^	2.75 (2.038)	3.13 (1.65)	*T*(98)=−1.007, *P*=0.32
SSS Disinhibition^a^	4.06 (2.25)	3.96 (2.44)	*T*<1
SSS Experience Seeking^a^	5.90 (1.79)	5.21 (2.021)	*T*(98)=1.83, *P*=0.071
SSS Thrill and Adventure Seeking^a^	7.58 (2.24)	5.27 (3.058)	*T*(85.59)=4.33, *P*<0.001
UPPS-P Sensation Seeking^a^	37.65 (5.66)	32.00 (8.60)	*T*(80.27)=3.85, *P*<0.001
UPPS-P Lack of Perseverance^a^	17.92 (3.68)	21.96 (4.80)	*T*(98)=−4.81, *P*<0.001
UPPS-P Lack of Premeditation^a^	20.88 (5.26)	21.04 (5.71)	*T*<1
UPPS-P Negative Urgency^a^	22.69 (5.15)	31.94 (6.99)	*T*(86.012)=−7.49, *P*<0.001
UPPS-P Positive Urgency^a^	21.15 (6.68)	26.17 (9.87)	*T*(81.66)=−2.95, *P*=0.004

Abbreviations: BAS, Behavioral Activation System Scales; BIC, Bayesian Information Criterion; BIS-11, Barratt Impulsiveness Scale; F, female; HAMA, Hamilton Anxiety Rating Scale; HAMD, Hamilton Rating Scale for Depression; M, male; MASQ, Mood and Anxiety Symptom Questionnaire; SHAPS, Snaith-Hamilton Pleasure Scale; SSS, Sensation Seeking Scale; STAI, State-Trait Anxiety Inventory; YMRS, Young Mania Rating Scale.

a Inclusion in the elastic net regression models as independent measures. Duplicate measures of the same factor were not included, for example, IQ was not included because of overlap with years of education, which was included; STAI state and HAMA were not included because of overlap with STAI-trait.

b Behavioral data: high performing participants only (*n*=91); BIC applies to behavioral model fit.

**Table 2:** Description of the elastic net and conventional regression model statistics

*Region*	*Elastic net predictors (numbers reflect exponents)*	*ISSc with covariates*	*Group with covariates*	*ISSc w/o covariates*	*Group w/o covariates*
Mean of all three regions	**BIS-11 M: 0.0040** **BIS-11 A: 0.015** BIS-11 NP: 0.0027 **BAS-FS: 0.0043** BAS-D: -0.0087 BAS-RR: -0.00023 **UPPS-P PU: 0.0037** **UPPS-P NU: 0.0062** Group: -0.13 Anxiety: -0.0029 Motion: 0.26	*t*=4.62, *P*<0.001	*t*=−3.49, *P*=0.001	*t*=5.23, *P*<0.001	*t*=−3.94, *P*<0.001
Left VLPFC	**UPPS-P PU: 0.0019** **UPPS-P NU: 0.0053**	*t*=3.31, *P*=0.001	*t*=−1.17, *P*=0.23	*t*=3.52, *P*=0.001	*t*=−1.040, *P*=-0.29
Left VS	**BIS-11 M: 0.0095** **BIS-11 A: 0.014** **UPPS-P PU: 0.0023** **UPPS-P NU: 0.00095** Group: -0.14 HAMD: -0.0034 Motion: 0.23	*t*=3.68, *P*<0.001	*t*=−3.81, *P*<0.001	*t*=4.36, *P*<0.001	*t*=−4.38, *P*<0.001
Right VS	**BAS-FS; 0.0023** Group: -0.033 Anxiety: -0.00030	*t*=3.51, *P*=0.001	*t*=−3.23, *P*=0.002	*t*=4.065, *P*<0.001	*t*=−3.90, *P*<0.001

Abbreviations: BAS, Behavioral Activation System Scales; BIS-11, Barratt Impulsivity Scale; HAMD, Hamilton Rating Scale for Depression; ISS, impulsive sensation seeking; ISSc, ISS subcomponents; VLPFC, vasolateral prefrontal cortex; VS, ventral striatum.

Scales contributing to the ISS composite are marked in bold. Such scales include the motor (M) and attentional (A) scales of the BIS-11, the Positive (PU) and Negative urgency (NU) scales of the UPPS-P, and the fun-seeking (FS) scale of the BAS. The third and fourth columns describe the effects of group and ISSc derived from a single multiple regression model, which also included gender, age, education and framewise displacement as covariates. The fifth and sixth columns describe the same model without those four covariates.
