# Supplementary material for: A pathway linking reward circuitry, impulsive sensation-seeking and risky decision-making in young adults: identifying neural markers for new interventions
Source: Transl Psychiatry. 2017 Apr 18;7(4):e1096–. doi: 10.1038/tp.2017.60 (PMC5416701; doi:10.1038/tp.2017.60)
Supplement: Supplementary Information [file tp201760x1.docx]

Methods

*Exclusion criteria*

Exclusion criteria were: history of head injury, neurological, pervasive developmental disorder or systemic medical disease (from medical records and report by each potential participant); cognitive impairment (Mini-Mental State Examination^1^ score<24, and premorbid NAART IQ^2^ estimate<85; visual disturbance (<20/40 Snellen visual acuity); left or mixed handedness (Annett criteria^3^); alcohol/substance abuse/dependence (including nicotine) and/or illicit substance use (except cannabis) over the last 3 months, determined by Structured Clinical Interview for DSM-5 (SCID)^4^ (and psychiatric records, if available). Lifetime/present cannabis use (non-abuse levels) was allowed, given its common usage in 18-25 year-olds^5^. Urine tests on the scanning day excluded individuals with current illicit substance use (except cannabis); salivary alcohol tests excluded individuals who are intoxicated on the scanning day. Additional exclusion criteria were MRI screening exclusion criteria, and positive pregnancy test for female individuals or self-reporting of pregnancy; and taking any psychotropic medication or medication combination for >2 weeks, and/or being medication free for less than 3 months prior to recent (2 weeks) medication. Having a previous history of seeking help for psychological distress, e.g., any emotional, behavioral or substance abuse/dependence problems, irrespective of having received a DSM diagnosis or not, was allowed in distressed individuals, as long as psychotropic medication, if previously prescribed, was no longer being taken, and the individual was free from such medication for a minimum of 6 months (or present medication taken for <2 weeks).

*Regions of interest*

Three regions of interest (ROI) were employed for statistical analysis of the BOLD images. The left and right VS were defined on the basis of activity derived from a previous study^6^. The left vlPFC ROI was constructed from several loci in this region from studies showing increased reward-related activity in bipolar disorder or individuals at high risk^6-10^. The co-ordinates of these studies were recorded and submitted to an activation likelihood estimation (ALE) meta-analysis^11^. The ALE algorithm convolved the peaks with a smooth Gaussian function, weighted by the sample size. The resulting cluster (peak voxel -45, 26, -8; 344 voxels: see figure S1) was thresholded at p<0.001 and used for subsequent analyses. Although this was not designed to be a valid meta-analysis, it provided the selection of co-ordinates of interest with a degree of independence from any particular prior study.


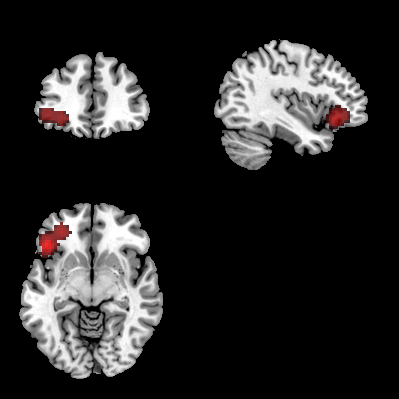


Figure S1: Left ventrolateral PFC region of interest

*Post scan risky decision making*

Following the scanning session, participants were asked to perform a decision making paradigm using the stimuli (cards) that they had seen during the reward paradigm in the scanner. To begin with, participants were given the following introduction to the task: *“On a series of trials, you will now be asked to choose between two options. One option will be one of the four cards you saw while you were doing the card guessing game. The presence of these cards might have affected whether you got $1 wins, 75 cent losses or nothing on a particular trial. In this part of the test, the cards have the same meaning, so a card that led to a higher chance of winning in the card guessing game would give the same chance of winning here. Likewise, cards that led to a higher chance of losing would give the same chance of losing, and so on”.* Participants were given a brief reminder of the cards and their associated outcomes, as they had been at the practice phase. Participants were then told the following: *“The other option to choose from will be a sure win or loss of a displayed amount. On each turn, you should decide between the two options - the card or the sure amount - picking the option you think would give the best return. Your choice will be marked on the screen, but you won't get any feedback about your choice.”* The card or ‘sure thing’ options were randomly displayed on different sides of the screen, and participants selected a given option by pressing a key that corresponded to the side of the option. A marker was displayed below the selected option, denoting its selection. Prior to the display of the options, there was a 1 second fixation cross, but no time limit was enforced in the paradigm, so individuals could take as long as they wanted to make a choice. As stated in the instructions, no feedback was given to the participant about their choice.

Choice behavior on the post-scan task was modeled using a two parameter model. The key parameter of interest was a ‘bias’ parameter, which reflected the relative (subjective) likelihood of wins versus losses. In addition, we included a parameter reflecting stochastic choice or temperature (‘beta’). Choice between the card and the sure thing option was modeled in terms of the expected value of each option, which determined choice via a softmax function:

$$P\left( card \right)= \frac{e^{\frac{Qcard}{\beta}}}{e^{\frac{Qcard}{\beta}}+e^{\frac{Qsure}{\beta}}}$$

Thus, the probability of selecting the card (P(card)) was a function of the expected values of the card (Qcard) and sure thing options (Qsure). The expected value of the card was calculated by multiplying the possible outcomes of the card (e.g. $1 and -$0.75 for the mixed card) by the probability of each outcome. This was objectively 0.5, but the bias parameter reflected an enhanced subjective probability of winning options compared to loss options i.e. an optimistic bias. A sigmoidal function was used to constrain this probability between 0 and 1, so a bias score of zero represents no bias. However, to enhance interpretability of the scores, we transformed the bias and beta scores to what they would be if they had not been transformed by a sigmoidal function. Thus, the bias scores slightly greater than 0.5 seen in most participants (main text: Table 1/Figure 2B) represent a slight optimistic bias, as 0.5 is the veridical probability of the card. Statistics were performed on the untransformed values however, although these were very similar to the transformed values.

The variational Bayes analysis (VBA) toolbox ^12^ was used for model fitting, with priors for each parameter set with a mean of zero and standard deviation of 0.5, and diagonal, cross-parameter priors set to zero (mean and standard deviation). A second model was constructed in the same way, but including a third parameter reflecting non-linear utility within the sub-$1 options (‘curve’). This model provided a better fit of the data, but not one that was accompanied by a superior BIC. Nevertheless, the bias and beta parameters estimated with this model were very similar to those estimated by the 2 parameter model, yielding similar relationships with the independent measures.

The advantage of the two parameter model is that it is a relatively efficient way to describe the data. However, it should be noted that the model does not allow us to distinguish accounts of the data based on variation in learning about the cards versus optimistic choice which is independent of learning. This is mostly due to the design of the choice paradigm: a model in which the bias parameter was determined by learning during the fMRI task generated similar data. Modelling also does not provide insight into ambiguity versus risk preference, which may independently influence choice of the cards over sure thing options.

Nine participants showed poor model fits, on both the 2 and 3 parameter models. These individuals formed a separate distribution, distinct from the other 91 participants, in terms of their model fits (free energy) and beta scores. Specifically, while 91 participants included showed model fits (free energy) between -22.8 and -40.2 within a roughly continuous distribution, the remaining 9 showed model fits between -44.1 and -47.5. Figure S2 displays the Bayesian Information Criterion (BIC) for all participants, also revealing the bimodal distribution. The distribution of beta scores was also bimodal, with a slightly more pronounced distinction between the two clusters. It is difficult to interpret data derived from poorly fitting models, and given the naturally separate distribution of poorly fitting participants, we chose to exclude their data for subsequent analysis. Three of the excluded 9 were controls, while 6 were distressed, but the difference was not significant (Fisher’s exact test p=0.30).


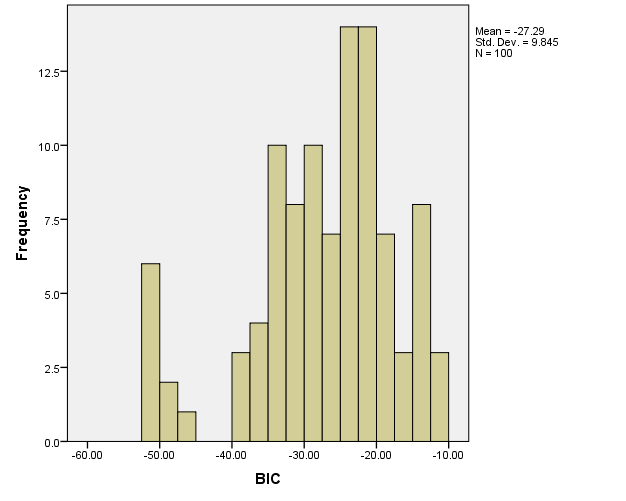


Figure S2: Distribution of BIC scores derived from model fitting, revealing a small cluster of individuals with poor fits (BIC around -50).

*Statistical approach (elastic net)*

To test our main aims, we used elastic net regression analyses, which has the advantage over conventional multiple regression models of allowing inclusion of a large number of correlated independent variables. Although other multivariate measures (PCA) applied to the same dataset yielded similar findings, we preferred the elastic net approach as it enforced a sparse solution, selecting a highly restricted number of measures. It is important to emphasize that the goal of the elastic net is somewhat different to conventional estimates of significance and effect size, insofar as it is focused on prediction – the capacity of the independent variables to predict the dependent variables. We followed these elastic net analyses with multiple regression models to determine the significance of ISS trait component and subcomponent-neural activity and ISS trait component-bias relationships in elastic net regression analyses. Only specific ISS subcomponents were positively correlated with activity in left vlPFC and bilateral VS during uncertain RE, and with bias, even after covarying for other symptoms and demographic variables.

The elastic net includes a parameter which balances the λ1 and λ2 penalty terms. This was set to 0.5^13^, though findings were largely insensitive to small changes to this value. 10-fold cross validation and 10 monte-carlo simulations were also performed for each model.

*Additional analyses*

Relationships between individual psychiatric diagnoses and RE-related activity in the three ROIs and bias were investigated. A marginal finding was observed, in which left VS RE-related activity was higher in individuals endorsing a lifetime mood disorder compared to those without (t(46)=2.079, p=0.043), but this did not survive statistical correction for multiple tests of the presence of different diagnoses. Otherwise, no significant findings were obtained.

| Diagnosis | Endorsed / Not Endorsed |
| --- | --- |
| Lifetime Mood disorder | 34/14 |
| Lifetime Bipolar disorder | 2/46 |
| Lifetime Unipolar Depression | 32/16 |
| Lifetime Anxiety Disorder | 29/19 |
| Lifetime Substance Use Disorder | 7/41 |
| Current Bipolar disorder | 0/48 |
| Current Major Depression | 12/32 |
| Current ADHD | 4/44 |

Table S1: Allocation of participants within the distressed group into diagnostic categories.

*Medication*

Three of the patients were medicated: one with Sertraline: 50mg/day, one with Citalopram: 10mg/day, and one with Lithium Orotate (10mg/day – dietary supplement). Non-parametric tests did not reveal any relationship between medication status and the primary measures of interest (p’s>0.075).

*Neuroimaging data analysis: Whole brain co-ordinates*

Table S2 and S3 describe the effects of ISSc and SSS ES, respectively, on RE-related wholebrain activity at the whole brain level. Exploratory analyses revealed no effects of ISSc or of SSS ES on uncertain outcome expectancy (OE) or reward prediction error (PE) contrasts at a corrected threshold (p<0.05 FWE peak level threshold). Table S4 describes the main effect of bias on RE-related wholebrain activity in participants with high ISSc (high and low ISSc subgroups defined by median split of ISSc scores). Table S5 describes the main effect of group on RE-related wholebrain activity. Table S6 describes the positive effect of ISSc on the contrast of possible win versus neutral expectancy, and possible loss versus neutral expectancy. Tables S7 and S8 describe the effect of the uncertain RE and OE regressors on wholebrain activity across all participants. The PE regressor-related wholebrain activity recapitulated previous findings, with highly significant activity (all T>5.15, peak level p<0.01 FWE) in ventral striatum, medial and left superior prefrontal cortex, posterior cingulate, left temporal lobe and thalamus.

| Region | T statistics (FWE peak level p<0.05) | MNI coordinates | Cluster volume |
| --- | --- | --- | --- |
| Left vlPFC, frontal operculum, central OFC, bilateral caudate | 5.61 (p=0.002)  4.85 (p=0.030)  4.80  4.68  4.61  4.57 | -34 26 -6  -14 36 -6  16 40 -6  -34 36 -4  8 18 0  -46 24 18 | 2316 |
| Right frontal operculum, central OFC, IFG | 4.26  4.23  3.50  3.49  3.49  3.44 | 16 34 6  30 34 0  30 46 10  42 32 14  24 40 16  36 26 -8 | 577 |
| Left inferior parietal lobule (IPS: hIP1, hIP2, hIP3) | 4.12  4.08  3.52 | -28 -60 32  -36 -52 44  -50 -40 44 | 380 |
| Right Angular Gyrus, Superior Occipital Gyrus | 4.05  3.75 | 32 -56 40  28 -68 30 | 269 |
| Right Caudate (body) | 4.51  4.32 | 14 4 26  16 12 26 | 267 |
| Right middle, inferior frontal gyrus | 4.40  3.57  3.49 | 38 6 40  36 6 28  36 16 26 | 260 |
| Left inferior frontal gyrus | 4.08 | -40 14 32 | 213 |
| Right middle occipital gyrus (hOc4la) | 3.99 | 38 -78 0 | 82 |
| Posterior Medial Frontal Cortex | 3.76 | -2 22 46 | 54 |
| Right anterior cingulate cortex | 3.55 | -12 38 12 | 28 |
| Corpus callosum | 3.57 | 6 -36 16 | 25 |
| Left Middle frontal gyrus | 3.55 | -30 8 44 | 25 |

Table S2: Positive effects of ISSc on RE-related wholebrain activity (p<0.001 cluster forming, 20 voxel threshold), including covariates. Statistics for findings which reach p<0.05 FWE peak level significance are marked in parentheses in the second column.

| Region | T statistics (FWE peak voxel p<0.05) | MNI coordinates | Cluster volume |
| --- | --- | --- | --- |
| Calcarine gyrus, Fusiform gyrus (hOc1, hOc2) | 5.23 (p=0.008)  5.04 (p=0.016)  4.63  3.89  3.87 | 26 -50 0  -6 -64 10  14 -72 6  28 -64 -6  -22 -62 12 | 1784 |
| Mid cingulate cortex | 4.50  4.24  4.21  4.12 | -2 -2 36  -4 6 38  4 0 34  10 10 38 | 892 |
| Premotor Cortex | 4.87 (p=0.029)  4.19  3.76  3.32 | 8 0 68  20 6 54  24 -2 62  2 -2 54 | 624 |
| Posterior Cingulate, Superior Parietal Lobule (5Ci) | 4.09 | 10 -34 42 | 289 |
| Right Superior Temporal Gyrus, Supramarginal Gyrus (OP1 - SII) | 3.90  3.48 | 52 -24 12  64 -18 18 | 284 |
| Right Superior Parietal Lobule (5L) | 3.75  3.65  3.55 | 18 -50 70  22 -48 54  16 -58 50 | 204 |
| Left Lingual / Fusiform Gyrus | 3.88  3.80 | -22 -48 -8  -28 -48 -6 | 173 |
| Left Superior Temporal Gyrus, Insula (Id1) | 3.68  3.61 | -44 -4 -4  -46 -6 -8 | 132 |
| Right Precentral Gyrus | 3.79  3.53 | 50 4 38  42 4 28 | 103 |
| Left Superior Parietal Lobule (7A) | 3.64  3.55 | -16 -70 48  -14 -74 40 | 101 |
| Superior Occipital Gyrus / Cuneus | 3.43  3.35  3.31  3.30 | 0 -86 32  6 -84 28  8 -78 26  12 -72 24 | 82 |
| Right Postcentral Gyrus (3a) | 3.72 | 32 -30 44 | 56 |
| Posterior Cingulate, Superior Parietal Lobule (5Ci) | 3.71 | -12 -32 38 | 42 |
| Left Lingual Gyrus (hcOc4v / FG1) | 3.54 | -24 -66 -12 | 39 |
| Right Cerebellum | 3.88  3.76  3.54 | 22 -32 -22  24 -36 -24  26 -38 -26 | 37 |
| Left Cerebellum | 3.55 | -28 -48 -26 | 30 |
| Right Superior Occipital Gyrus | 3.38 | 26 -74 28 | 22 |
| Left Superior Frontal Gyrus | 3.37 | -20 2 64 | 21 |

Table S3: Positive effects of SSS ES on RE-related wholebrain activity (p<0.001 cluster forming, 20 voxel threshold), including covariates. Statistics for findings which reach p<0.05 FWE peak level significance are marked in parentheses in the second column.

| Region | T statistics (FWE peak voxel p<0.05) | MNI coordinates | Cluster volume |
| --- | --- | --- | --- |
| Anterior Cingulate Cortex | 6.17 (p=0.010)  5.29 | -4 18 20  6 20 20 | 255 |
| Left Premotor Cortex | 4.75 | -16 -2 64 | 60 |

Table S4: Positive association between bias and RE related activity in participants with high ISSc (z score > 0) across all subjects (p<0.001 cluster forming, 20 voxel threshold), including motion and demographic variables as covariates. Statistics for findings which reach p<0.05 FWE peak level significance are marked in parentheses in the second column. No significant associations were seen at this threshold in low ISSc participants.

| Region | T statistics (FWE peak voxel p<0.05) | MNI coordinates | Cluster volume |
| --- | --- | --- | --- |
| Left frontal operculum | 5.27 (p=0.007)  5.00 (p<0.05)  3.66  3.54  3.34 | -30 26 -6  -28 22 -10  -18 20 -12  -38 16 -4  -32 18 4 | 423 |
| Right vlPFC/ frontal operculum | 4.22 | 34 26 -8 | 125 |
| Left Caudate | 4.06  3.21 | -6 14 -2  -12 18 -8 | 111 |
| Anterior Cingulate Cortex | 3.62  3.31 | -6 40 18  4 44 18 | 58 |
| Left thalamus (parietal/motor/somatosensory regions) | 3.77 | -16 -24 0 | 40 |
| Right Caudate | 3.62 | 8 16 -2 | 39 |
| Left Central OFC | 3.60 | -18 36 -14 | 22 |

Table S5: Effect of group (controls > distressed) on RE-related wholebrain activity (p<0.001 cluster forming, 20 voxel threshold), including covariates. Statistics for findings which reach p<0.05 FWE peak level significance are marked in parentheses in the second column.

| Region | T statistics (FWE peak voxel) | MNI coordinates | Cluster volume |
| --- | --- | --- | --- |
| Left Inferior Parietal Lobule (hIP1, hIP2, hIP3) | 4.65 (p=0.057)  4.58  4.45 | -36 -50 44  -30 -58 40  -46 -44 54 | 814 |
| Right Inferior Parietal Lobule (BA 2, hIP2, PFt) | 4.22  3.66  3.60 | 48 -34 52  42 -46 50  40 -44 40 | 350 |
| Left Precentral Gyrus | 3.62 | -44 2 36 | 60 |
| Left Inferior Frontal Gyrus | 4.17 | -48 36 14 | 59 |
| Right Inferior Frontal Gyrus | 3.71  3.47 | 44 18 10  48 26 14 | 50 |
| Right Superior Frontal Gyrus | 4.06  3.36 | 18 -14 72  28 -6 66 | 48 |
| Left Middle Frontal Gyrus | 3.93 | -28 4 62 | 41 |
| Left Inferior Frontal Gyrus (BA44, 45) | 3.82 | -54 22 18 | 29 |
| Right Insula | 3.48 | 34 20 -4 | 26 |
| Right Middle Frontal Gyrus | 3.72 | 38 -4 58 | 20 |

Table S6: Positive effect of ISSc on the contrast of possible win versus neutral expectancy (p<0.001, 20 voxels). Statistics for findings which approach p<0.05 FWE peak level significance are marked in parentheses in the second column.

| Region | T statistics (FWE peak voxel p<0.05) | MNI coordinates | Cluster volume |
| --- | --- | --- | --- |
| Anterior Cingulate Cortex, Caudate, Putamen | 5.87 (p=0.001)  5.47 (p=0.003)  4.86 (p=0.028)  4.85 (p=0.028)  4.70 (p=0.047)  4.67 | 2 32 16  12 14 -8  -18 8 -10  -16 12 -10  -6 2 -6  -4 42 4 | 2350 |
| Visual cortex (Lingual/Calcarine gyrus) | 8.10 (p<0.001)  6.87 (p<0.001)  6.19 (p<0.001)  4.40  4.34 | -22 -86 -14  6 -94 8  -6 -94 12  20 -82 -10  14 -86 -10 | 2010 |
| Right Caudate (body) | 4.58  4.37  3.82  3.67 | 28 -16 26  10 -6 26  0 -6 34  6 -10 32 | 409 |
| Cerebellum (Lobule IX) | 3.83  3.69 | 6 -44 -34  -4 -48 -36 | 71 |
| Cerebellar Vermis | 4.02 | 6 -36 -6 | 42 |
| Right Thalamus (temporal, visual subregions) | 3.54  3.46 | 26 -30 0  24 -28 -8 | 35 |
| Right Inferior Frontal Gyrus | -4.41  -4.32  -4.13  -4.11 | 46 24 28  44 18 36  34 10 32  36 12 34 | 591 |
| Superior medial gyrus | -4.14 | -4 28 48 | 221 |
| Left Inferior Frontal Gyrus | -3.62  -3.54 | -46 18 30  -40 26 22 | 126 |
| Right Inferior Temporal Gyrus (hOc4la, FG2) | -4.16 | 46 -74 -8 | 96 |
| Left Inferior Frontal Gyrus | -3.76 | -42 48 -10 | 20 |

Table S7: Main effect of uncertain RE (both positive and negative) on wholebrain activity across all subjects (p<0.001, 20 voxels). Statistics for findings which reach p<0.05 FWE peak level significance are marked in parentheses in the second column. Motion (framewise displacement) was included as a covariate of no interest.

| Region | T statistics (FWE peak voxel p<0.05) | MNI coordinates | Cluster volume |
| --- | --- | --- | --- |
| Bilateral Middle, Superior, Inferior Occipital, Fusiform Gyrus (hOc4lp/la/v), FG2 | 14.31 (p<0.001)  13.76 (p<0.001)  13.64 (p<0.001)  11.29 (p<0.001)  10.01 (p<0.001) | 44 -74 -8  -34 -84 -4  -32 -88 6  32 -84 14  -24 -76 -10 | 12567 |
| Bilateral Thalamus | 4.96 (p=0.019)  4.61  4.04 | -20 -30 -4  -4 -30 -6  6 -28 -4 | 285 |
| Right Thalamus | 4.31 | 22 -28 0 | 87 |
| Right Prefrontal Cortex (dorsal, inferior, superior, ventral), right putamen. | -6.59 (p<0.001)  -6.17 (p<0.001)  -6.12 (p<0.001)  -5.70 (p=0.001)  -5.62 (p=0.002)  -5.58 (p=0.002) | 26 40 42  20 36 -12  22 38 -10  38 32 44  30 12 32  24 58 8 | 6666 |
| Left Prefrontal Cortex (middle, superior) | -5.98 (p<0.001)  -4.94 (p=0.020)  -4.93 (p<0.05)  -4.48  -4.40  -4.21 | -26 40 42  -34 38 38  -36 36 36  -16 30 28  -20 34 24  -30 30 24 | 1506 |
| Right Inferior Parietal Lobule (PGp, PGa, PFm) | -5.79 (p=0.001)  -5.31 (p=0.005) | 54 -60 26  52 -68 32 | 518 |
| Left Central OFC | -4.69 (p=0.046)  -4.68 (p=0.049) | -26 42 -10  -18 38 -12 | 268 |
| Left Superior Frontal Gyrus | -4.64  -3.80 | -20 54 6  -34 54 12 | 247 |
| Left Putamen | -3.87  -3.70 | -24 10 -4  -26 -2 -6 | 130 |
| Right Superior, Middle Frontal Gyrus (TE 3) | -3.80  -3.47 | 66 -30 4  66 -42 6 | 91 |
| Posterior Cingulate Cortex | -3.61  -3.58 | 16 -42 36  4 -42 38 | 64 |

Table S8: Effect of uncertain Outcome Expectancy (OE), across all participants on wholebrain activity across all subjects (p<0.001, 20 voxels). Statistics for findings which reach p<0.05 FWE peak level significance are marked in parentheses in the second column. Motion (framewise displacement) was included as a covariate of no interest.

|  | BAS-FS | BIS-11 Att | BIS-11 M | UPPS-P NU | UPPS-P PU |
| --- | --- | --- | --- | --- | --- |
| BAS-FS |  |  |  |  |  |
| BIS-11 Att | 0.22 (0.027) |  |  |  |  |
| BIS-11 M | 0.61 (<0.001) | 0.22 (0.030) |  |  |  |
| UPPS-P NU | 0.19 (0.060) | 0.53 (<0.001) | 0.22 (0.027) |  |  |
| UPPS-P PU | 0.37 (<0.001) | 0.36 (<0.001) | 0.36 (<0.001) | 0.65 (<0.001) |  |

Table S9: Table to describe inter-correlations (Pearson’s R, p values in parentheses) between the five ISS scales identified with the elastic net analysis.

References

1. Folstein MF, Folstein SE, McHugh PR. "Mini-mental state". A practical method for grading the cognitive state of patients for the clinician. *J Psychiatr Res* 1975; **12**(3)**:** 189-198.

2. Blair JR, Spreen O. Predicting premorbid IQ: A revision of the national adult reading test. *Clinical Neuropsychologist* 1989; **3**(2)**:** 129-136.

3. Annett M. A classification of hand preference by association analysis. *Br J Psychol* 1970; **61**(3)**:** 303-321.

4. First MB, Williams JBW, Karg RS, Spitzer RL. *Structured Clinical Interview for DSM-5—Research Version (SCID-5 for DSM-5, Research Version; SCID-5-RV).* . American Psychiatric Association: Arlington, VA., 2015.

5. SAMHSA. 2010 National Survey on Drug Use and Health. Dept. of Health and Human Services, Substance Abuse and Mental Health Services Administration, Office of Applied Studies Rockville, MD, 2011.

6. Chase HW, Nusslock R, Almeida JR, Forbes EE, Labarbara EJ, Phillips ML. Dissociable patterns of abnormal frontal cortical activation during anticipation of an uncertain reward or loss in bipolar versus major depression. *Bipolar Disord* 2013.

7. Bermpohl F, Kahnt T, Dalanay U, Hagele C, Sajonz B, Wegner T *et al.* Altered representation of expected value in the orbitofrontal cortex in mania. *Hum Brain Mapp* 2010; **31**(7)**:** 958-969.

8. Caseras X, Lawrence NS, Murphy K, Wise RG, Phillips ML. Ventral Striatum Activity in Response to Reward: Differences Between Bipolar I and II Disorders. *Am J Psychiatry* 2013.

9. Nusslock R, Almeida JR, Forbes EE, Versace A, Frank E, Labarbara EJ *et al.* Waiting to win: elevated striatal and orbitofrontal cortical activity during reward anticipation in euthymic bipolar disorder adults. *Bipolar Disord* 2012; **14**(3)**:** 249-260.

10. Singh MK, Kelley RG, Howe ME, Reiss AL, Gotlib IH, Chang KD. Reward processing in healthy offspring of parents with bipolar disorder. *JAMA Psychiatry* 2014; **71**(10)**:** 1148-1156.

11. Eickhoff SB, Laird AR, Grefkes C, Wang LE, Zilles K, Fox PT. Coordinate-based activation likelihood estimation meta-analysis of neuroimaging data: a random-effects approach based on empirical estimates of spatial uncertainty. *Hum Brain Mapp* 2009; **30**(9)**:** 2907-2926.

12. Daunizeau J, Adam V, Rigoux L. VBA: a probabilistic treatment of nonlinear models for neurobiological and behavioural data. *PLoS Comput Biol* 2014; **10**(1)**:** e1003441.

13. Zou H, Hastie T. Regularization and variable selection via the elastic net. *J Roy Stat Soc B* 2005; **67:** 301-320.
